# The “Endless Trip” among the NPS Users: Psychopathology and Psychopharmacology in the Hallucinogen-Persisting Perception Disorder. A Systematic Review

**DOI:** 10.3389/fpsyt.2017.00240

**Published:** 2017-11-20

**Authors:** Laura Orsolini, Gabriele Duccio Papanti, Domenico De Berardis, Amira Guirguis, John Martin Corkery, Fabrizio Schifano

**Affiliations:** ^1^Psychopharmacology, Drug Misuse and Novel Psychoactive Substances Research Unit, School of Life and Medical Sciences, University of Hertfordshire, Hatfield, United Kingdom; ^2^Neomesia Mental Health, Villa Jolanda Hospital, Jesi, Italy; ^3^Polyedra, Teramo, Italy; ^4^NHS, Department of Mental Health, Psychiatric Service of Diagnosis and Treatment, Hospital “G. Mazzini”, Teramo, Italy; ^5^Department of Neuroscience, Imaging and Clinical Science, Chair of Psychiatry, University “G. D’Annunzio”, Chieti, Italy

**Keywords:** hallucinogen-persisting perception disorder, novel psychoactive substances, hallucinogens, hallucinations, flashbacks, palinopsia

## Abstract

Hallucinogen-persisting perception disorder (HPPD) is a syndrome characterized by prolonged or reoccurring perceptual symptoms, reminiscent of acute hallucinogen effects. HPPD was associated with a broader range of LSD (lysergic acid diethylamide)-like substances, cannabis, methylenedioxymethamphetamine (MDMA), psilocybin, mescaline, and psychostimulants. The recent emergence of novel psychoactive substances (NPS) posed a critical concern regarding the new onset of psychiatric symptoms/syndromes, including cases of HPPD. Symptomatology mainly comprises visual disorders (i.e., geometric pseudo-hallucinations, haloes, flashes of colors/lights, motion-perception deficits, afterimages, micropsia, more acute awareness of floaters, etc.), even though depressive symptoms and thought disorders may be comorbidly present. Although HPPD was first described in 1954, it was just established as a fully syndrome in 2000, with the revised fourth version of the Diagnostic and Statistical Manual of Mental Disorders (DSM-IV-TR). HPPD neural substrates, risk factors, and aetiopathogenesys still largely remain unknown and under investigation, and many questions about its pharmacological targets remain unanswered too. A critical mini review on psychopathological bases, etiological hypothesis, and psychopharmacological approaches toward HPPD, including the association with some novel substances, are provided here, by means of a literature search on PubMed/Medline, Google Scholar, and Scopus databases without time restrictions, by using a specific set of keywords. Pharmacological and clinical issues are considered, and practical psychopharmacological recommendations and clinical guidelines are suggested.

## Introduction

Hallucinogen-persisting perception disorder (HPPD) is a long-lasting and potentially permanent syndrome characterized by a spontaneous recurrence of perceptual/visual disturbances which are reminiscent of those generated while a subject was intoxicated with hallucinogens. According to the Fifth Version of Diagnostic and Statistical Manual of Mental Disorders (DSM-5), HPPD is defined as the following criteria (1):

(A) following cessation of use of a hallucinogen, the reexperiencing of one or more of the perceptual symptoms that were experienced while intoxicated with the hallucinogens (e.g., geometric hallucinations, false perceptions of movement in the peripheral visual fields, flashes of color, intensified colors, trial images of moving objects, positive after images, haloes around objects, macropsia, and micropsia);
(B) the symptoms in criterion (A) cause clinically significant distress or impairment in social, occupational, or other important areas of functioning;
(C) the symptoms are not due to a general medical condition (e.g., anatomical lesions and infections of the brain, visual epilepsies) and are not better accounted for by another mental disorder (e.g., delirium, dementia, schizophrenia) or hypnopompic hallucinations.

Before diagnosing an HPPD, post-traumatic stress disorder, depersonalization, derealization, and hallucinogen-induced psychotic mood or anxiety disorders should be excluded (2). Moreover, other causes of visual disturbances should be investigated and excluded, such as anatomical lesions, brain infections, epilepsy, schizophrenia, delirium state, or hypnopompic hallucinations (2). Furthermore, an association between the first intake, frequency, and quantity of drug taken and the likelihood of developing an HPPD has not been demonstrated, as has been the onset of the disorder following a single hallucinogenic experience (3).

### Epidemiology

Overall, prevalence of HPPD has been generally considered low (2). However, limited publications suggested that chronic visual disturbances may be relatively common among hallucinogens’ users. It has often been assumed that HPPD may be a severe clinical manifestation of the drug-induced visual changes (3–5). While the probability of flashbacks occurring in the wake of hallucinogen use may vary from 5 to 50% among hallucinogens’ users (6, 7), the probability of an HPPD being manifested is lower (3).

### Historical Background

Hallucinogen-persisting perception disorder was first described in 1954 (8). Subsequent observations have been then described (3, 4, 8–12). Horowitz (10) first introduced the term *flashbacks*, referring to recurrent and spontaneous perceptual distortions and unbidden images. When these “flashbacks” present as recurrent, but without a current acute, or chronic hallucinogen intake, the disturbance is referred to as HPPD. Horowitz (10) classified also three types of visual flashbacks: (a) *perceptual distortions* (e.g., seeing haloes around objects); (b) *heightened imagery* (e.g., visual experiences as much more vivid and dominant in one’s thoughts); and (c) *recurrent unbidden* images (e.g., subjects see objects that are not there). HPPD has been introduced under the diagnosis of *Post- hallucinogen Perception Disorder* in 1987 within the DSM-III-R (13). Subsequently, the DSM-IV-TR (14) recognized the syndrome as *Hallucinogen-Persisting Perception Disorder (Flashbacks)* (code 292.89) (15). The disorder was confirmed as nosological entity as well in the DSM-5 (1).

### Phenomenology

Hallucinogen-persisting perception disorder is characterized by a plethora of visual disturbances (e.g., geometric imagery, afterimages, false perceptions of movement in the peripheral fields, flashes of light, etc.) (3) (Table 1), including pseudohallucinations. It has been also associated with a LSD (lysergic acid diethylamide)-like dysphoria, panic attacks, and depressive symptomatology. Visual disturbances may be episodic, stress or substance-induced, or persistent. However, the episodes may last for 5 years or more and the symptomatology is disliked by patients (10). While a *flashback* is usually reported to be infrequent and episodic, HPPD is usually persisting and long-lasting. Moreover, some HPPD subjects report that adaptation to the dark takes significantly longer compared with the general population (16). Moreover, HPPD has been associated with abnormal results for tests of visual function, suggesting disinhibition in the processing of visual information (4, 16). The subject does not develop any paranoid misinterpretation related to and does not believe that their own visual hallucinogenic experiences currently occur (3). Therefore, it has been supposed that there is involvement of the primary visual cortex, the first cortical area responsible for geometric processing of visual input (17). Further data coming from clinical, psychological, and neurophysiological sources may suggest specific physiological changes in the visual system function implicated in the onset of hallucinations after the intake of psychedelics/hallucinogens (5). Table 1 summarizes all clinical and psychopathological characteristics associated with an HPPD.

**Table 1:** Main clinical and psychopathological characteristics in HPPD.

Psychopathological and clinical features	Description
Teleopsia	Objects are perceived much further away than they actually are

Pelopsia	Objects are perceived nearer than their actual size

Macropsia	Objects are perceived larger than their actual size

Micropsia	Objects are perceived smaller than their actual size

Criticism/egodystonic psychosis	Patient manifests criticism toward own thoughts and perceptual disturbances, as well as experiencing perceptual disorders perceived as inconsistent with one’s self concept or ego state

Depersonalization	A state in which some individual feels that either he/she him/herself or the outside world is unreal

Derealization	A state in which an individual feels a detachment within the self-regarding one’s mind or body or being a detached observer of oneself (e.g., Feeling like being inside a transparent bubble)

Feeling of body being light or heavy	

Visual trailing	Transient disturbance of visual motion perception of unknown origin (i.e., subject perceives a series of discrete stationary images trailing in the wake of otherwise normally moving objects)

Haloes around objects	A geometric shape, usually in the form of a disk, circle, ring, or rayed structure around an object really present

Afterimages/palinopsia	An image that continues to appear in one’s visual field after the exposure to the original image has ceased

Other visual disturbances	Flashes of color
	Intensified colors
	Colored images
	Geometric imagery
	False perception of movement of images in the peripheral-field

*HPPD, hallucinogen-persisting perception disorder*.

### Aetiopathogenesys

The pathogenesis of HPPD is currently unknown, even though it has been frequently reported to be associated with the intake of LSD (4, 16, 41). However, HPPD has also been reported following the consumption of all substances with hallucinogenic properties which possess pharmacological and clinical effects resembling those experienced with LSD by serotoninergic 5-HT2A (42), such as cannabis (18, 43, 44), 3,4-methylenedioxymethamphetamine (MDMA, aka “ecstasy”) (45–47), and the recently marketed novel psychoactive substances (NPS). In fact, the advent of NPS facilitated the onset of new psychopathologies and new clinical manifestations, including cases of HPPD, particularly following the intake of synthetic cannabinoids (SCs) and other new synthetic psychedelics and hallucinogens, which facilitated the reoccurrence of this disorder, by posing a new clinical concern to clinicians (19, 20, 48, 49).

### Types of HPPD

Two types of HPPD have been proposed here, accordingly to Lev-Ran (40). *Type-1 HPPD*, consistent with the definition of flashback provided by the ICD-10 (50), is characterized by brief reexperiences of altered perception, mood, and/or consciousness, as previously experienced during a hallucinogenic intoxication. Symptomatology may be pleasurable and even controllable. They may appear days to months after the hallucinogen-induced experience. The subject is usually aware of the unreality of their own experience. The perception of time may be altered. Visual perceptions usually comprise perceived increased color intensity, dimensionality, vibrancy, illusory changes, and movements of a perceived object. *Type-1 HPPD* comprises the *flashbacks* definition, while *type-2 HPPD* has been used to indicate the HPPD definition elaborated by Abraham (3) as well proposed in the DSM-5. The symptoms usually include palinopsia (afterimages effects), the occurrence of haloes, trails, akinetopsia, visual snows, etc. Sounds and other perceptions are usually not affected. Visual phenomena have been reported to be uncontrollable and disturbing. Symptomatology may be accompanied by depersonalization, derealization, anxiety, and depression (3).

The present systematic mini review aims at providing an overview of HPPD, by specifically focusing on both clinical manifestations and psychopharmacological approaches, in general, and among NPS users.

## Materials and Methods

### Search Sources and Strategies

A critical mini review was conducted, following the methods recommended by the Cochrane Collaboration (51) and the Preferred Reporting Items for Systematic Reviews and Meta-Analyses (PRISMA) guidelines (52). Searches were carried out by using PubMed/Medline, Google Scholar, and Scopus. We combined the search strategy of free text terms and exploded MESH headings for the topics of HPPD and NPS as follows: ((*Hallucinogen Persisting Perception Disorder [*Title/Abstract] OR *HPPD [*Title/Abstract])) OR ((*Hallucinogen Persisting Perception Disorder [*Title/Abstract] OR *HPPD [*Title/Abstract])) and ((*novel psychoactive substances* [Title/Abstract]) OR (*NPS*[Title/Abstract])). All articles through August 15, 2017 without time restriction were selected. In addition, the authors performed further secondary searches by using the reference listing of all eligible papers.

### Study Selection

We considered studies about HPPD, and whenever available, evaluating the relationship between HPPD and NPS. The authors examined all titles/abstracts and obtained full texts of potentially relevant papers. Two reviewers (LO and DP), independently and in duplicate, read the papers and selected papers according to the inclusion criteria. Duplicate publications were excluded. All the articles identified by the data sources, reporting original data related to HPPD in general and, more specifically, among NPS users, were considered in the present review. All experimental and observational study designs, including case reports and case series, were included as limited data have been published so far. Narrative and systematic reviews, letters to the editor, and book chapters were excluded, even though they were used for retrieving further secondary searches. To be included in the present review, studies were required to meet the following criteria: (a) empirical and peer-reviewed study; (b) at least an abstract with estimates and/or full results available/complete; (c) investigate HPPD in general and more specifically among NPS users; (d) human studies; and (e) provide data on psychopathological features and/or psychopharmacological treatments in these cases.

### Data Extraction and Management

LO and DP independently extracted the data on participant characteristics, intervention details, and outcomes measures. Disagreements were resolved by discussion and consensus with a third member of the team (DDB). Data were collected using an *ad hoc* developed data extraction spreadsheet.

### Characteristics of Included Studies

**Figure 1:**
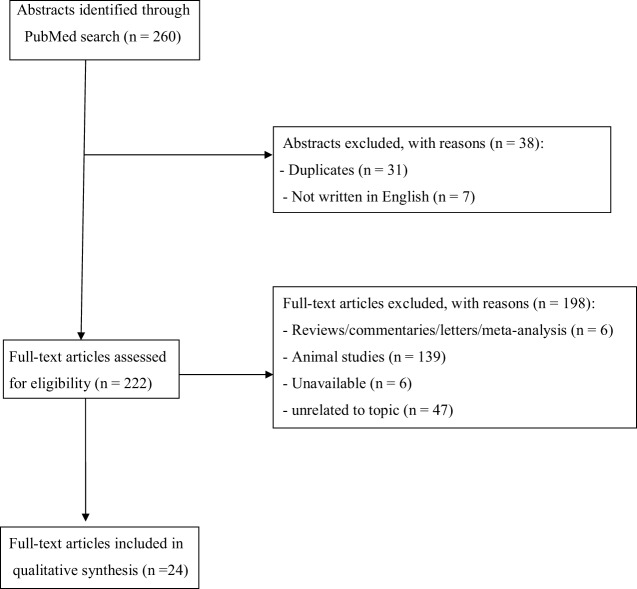
Selection of retrieved studies.

The set of keywords initially generated 260 results (Figure 1). A total of 31 papers were excluded because of duplicates; 7 papers were excluded because they did not provide relevant data useful for the aims of our papers (due to the lack of an English abstract). Of the remaining 222 studies, further 186 studies were excluded because they did not meet the inclusion criteria or because they were non-human studies. Of the remaining 36 papers, 6 papers were excluded because they were reviews, letters to editors, or metanalyses; however, 6 papers were not included here due to the lack of an available full text or an abstract useful for collecting relevant data. Finally, a total of 24 papers were included and accounted for in our analysis. Table 2 shows the main characteristics (study design, sample size, main outcomes, and findings) of all studies reviewed here.

**Table 2:** Summary of all included studies.

Study	Study design	Sample characteristics	Substance implicated	Psychopharmacological treatment (dosage)	Summary of findings
(3)	Observational study	21 HPPD	LSD	BZDs (N/A)	An improvement was observed among HPPD subjects following the use of BDZs; while phenothiazine worsened HPPD
				Phenothiazines (N/A)	

(18)	Case report	1 M, 18 years, student with a history of anxiety disorder	Cannabis and psilocybin	Amisulpride (100 mg daily)	Combination of risperidone and sertraline ameliorated HPPD symptomatology after 6 months of treatment
				Olanzapine (5 mg daily)	
				Risperidone (2 mg daily)	
				Sertraline (150 mg daily)	

(19)	Case reports	2 HPPD (DSM-IV-TR criteria): M, 26 years, college student who developed HPPD with recurrent panic attacks after discontinuation of SC intake M, 24 yr, who developed HPPD experienced with anxiety features after discontinuation of SC intake	SC	Clonazepam (1 mg/daily)	Clonazepam improved HPPD symptomatology

(20)	Case report	M, 18 years, with a history of heavy daily use of cannabis and SC who experienced HPPD	SC	Clonazepam (6 mg/daily)	For 3 years after SC consumption, the patient occasionally reexperienced the same symptoms developed during acute intoxication. These symptoms appeared during heavy cannabis consumption or in periods of boredom and inactivity

(21)	Case series	3 HPPD (DSM-IV criteria): Case 1: F (first LSD usage: 14 years; at 21 years, first acute onset of an LSD-like euphoria and persistent visual distortions, e.g., trails of objects, particles in air, round objects) Case 2: M, 22 years, college student (first LSD usage: 15–18 years; at 20 years, first acute onset of persistent visual symptoms of afterimages, trailing of stimuli, orange/blue haloes around objects) Case 3: M, 40 years, married, builder (first LSD usage: 18 years; at 18 years, first acute onset of dots on a blank wall, intensification of lights, trails of his hand, anxiety, depression)	LSD	RIS: 2–3 mg/daily 1–6 mg/daily 1–2 mg/daily	RIS worsened LSD-like panic and visual symptoms

(22)	Case series	2 HPPD (DSM-IV criteria)	LSD	Naltrexone (50 mg/daily)	Naltrexone caused a dramatic improvement in HPPD symptomatology. The remission was sustained also after discontinuation of naltrexone

(23)	Case report	M, 22 years who developed HPPD after an 8-month history of LSD abuse	LSD	Sertraline (100 mg/daily)	Sertraline determined initially an exacerbation of HPPD symptomatology, then it attenuated symptoms after 1 month’s administration

(24)	Observational study	8 HPPD	LSD	Clonidine (0.025 mg for 3 times/daily) for 2 months	Clonidine may alleviate LSD-related flashbacks

(25)	Case series	2 HPPD outpatients	LSD	Clonazepam	Clonazepam was efficacious in reducing HPPD symptomatology

(26)	Case report	1 HPPD with comorbid MDE	MDMA, LSD, and cannabis	Reboxetine (6 mg/daily)	Reboxetine did not exacerbates visual disturbances either recurrence of depressive features

(27)	Observational study	16 HPPD with anxiety features	LSD	Clonazepam (2 mg/daily)	Clonazepam was efficacious in attenuating both anxiety and HPPD symptomatology

(28)	Case report	F, 33 years with HPPD	LSD	Sertraline (200 mg daily)	Lamotrigine reduced almost completely visual disturbances of HPPD
				Citalopram (20–30 mg daily)	
				Fluoxetine (20 mg daily)	
				Risperidone (0.5–1 mg daily)	
				Lamotrigine (100–200 mg daily)	

(29)	Case report	M, 36 years with HPPD	LSD, cannabis, alcohol, cocaine	Clonidine	Clonidine did not improve symptomatology; while lamotrigine was associated with a significant symptomatology improvement
				Lamotrigine (200 mg/daily)	

(30)	Case report	F, 38 years with HPPD (DSM-5 criteria)	LSD	Risperidone (0.5 mg/daily)	Significant reduction in the frequency and intensity of panic attacks and perceptual disturbances within 3–4 weeks with low dosages of risperidone

(31)	Case report	M, 30 years, presented to the emergency department after surviving two subsequent suicide attempts by hanging, with a previous history of bipolar disorder and who developed HPPD	Cannabis	Citalopram (40 mg/daily)	Patient poorly responded to treatment and was found to have committed suicide
			LSD	Lamotrigine (50 mg/daily)	
			PCP	Mirtazapine (15 mg/daily)	
			Cocaine		

(32)	Web-based survey	626 hallucinogens’ users	Cannabis	N/A	Long-term perceptual disturbances were mainly reported among LSD users
			MDMA		
			Psilocybin		
			LSD		
			Ketamine		

(33)	Web-based survey	3139 hallucinogens’ users	Several hallucinogens (including cannabis, MDMA, psilocybin, LSD, ketamine, *Salvia divinorum*)	N/A	LSD appeared to be the most robust predictor of HPPD

(34)	Case reports	2 HPPD (DSM-5 criteria): M, 24 years, university student F, 25 years, university student	LSD	N/A	Both cases reported the appearance of visual disturbances that were not originally experienced during LSD intoxication

(35)	Case–control study	12 inpatients with schizophrenia and HPPD vs. 14 inpatients with schizophrenia without HPPD (DSM-IV-TR criteria)	LSD	N/A	No significant differences have been found between two groups in sociodemographic and clinical features. Individuals with schizophrenia and HPPD reported the ability to identify specific precursory cues for the appearance of HPPD-associated perceptual disturbances
			Cannabis		
			MDMA		

(36)	Case–control study	4 HC vs. 1 M, 23 years, HPPD patient	Cannabis	N/A	Cannabinoids may have a direct effect on the retina and retinal pigment epithelium function which may be involved in perceptual disturbances experienced in cannabis-induced HPPD

(37)	Case–control study	37 inpatients with schizophrenia and HPPD vs. 43 inpatients with schizophrenia without HPPD (DSM-IV-TR criteria)	LSD	N/A	No significant differences found between two groups in sociodemographic features. Individuals with schizophrenia and HPPD reported lower general psychopathology and negative symptoms scores compared with individuals without HPPD

(38)	Case report	M, 26 years, university student who developed AIWS and HPPD (DSM-5 criteria)	LSD	N/A	The patient refused any psychotropic treatment and after 1 year of psychiatric follow-up visual disturbances completely disappeared
			Cannabis		
			Alcohol		

(39)	Survey	23 out of 67 completed the survey (2 HC; 19 who reported persisting perceptual disturbances triggered or worsened by past drug use) 6 out of 19 with co- diagnosis of HPPD, 3 with persistent migraine aura, 2 psychotic disorders, 1 PTSD and 3 anxiety disorder, 2 depression, 2 hypochondriasis and 3 dissociative disorders HPPD	Various hallucinogenic and non-hallucinogenic drugs	N/A	Many perceptual symptoms reported were not first experienced while intoxicated and are partially associated with pre-existing psychiatric comorbidity

(40)	Observational study	40 patients who sought psychiatric consultation for SUD with a previous LSD intake who developed HPPD	LSD	N/A	Subjects with type-2 HPPD significantly more likely reported lifetime use of SC, stimulants and inhalants than type-1 HPPD (who reported more likely alcohol)

*HPPD, hallucinogen-persisting perception disorder; DSM, Diagnostic and Statistical Manual; F, female; M, male; LSD, lysergic acid diethylamide; yr, years; RIS, risperidone; HC, healthy controls; MDE, major depressive episode; SC, synthetic cannabinoids; PCP, phencyclidine; SUD, substance use disorders; N/A, not available; AIWS, Alice in Wonderland Syndrome*.

## Results

### Studies on Psychopharmacotherapy of HPPD

An observational study recruited 21 HPPD subjects who were treated with benzodiazepines and/or phenothiazines (3). Among subjects receiving benzodiazepines, eight out of nine reported a reduction in intensity/frequency of visual disorders. Most (11 out of 12) phenothiazine-treated subjects described an exacerbation of HPPD (3). A case series reported three cases of HPPD, treated with risperidone, who presented a worsening of visual perceptions and panic symptomatology (21). Lerner et al. (22) described two LSD-induced HPPD male subjects who reported an improvement of symptomatology following naltrexone (50 mg daily) treatment (22). Another case report described a 22-old-year male who developed HPPD after 8-month discontinuation of LSD (22). The subject significantly improved after sertraline (100 mg/daily) treatment (23). An open-label pilot study recruited eight HPPD drug-free patients who were consecutively treated with 0.025 mg of clonidine, three times a day, for 2 months, in order to evaluate the efficacy of drug in treating persistent visual disturbances associated with the intake of LSD (24). LSD-related flashbacks demonstrated a good response to clonidine (24). A study described two HPPD outpatients who efficaciously responded to clonazepam (25). An HPPD patient with a comorbid depressive symptomatology and a prior history of cannabis, ecstasy and LSD abuse, clinically responded to 6 mg daily of reboxetine (26). An open-label study recruited 16 drug-free patients affected with HPPD with anxiety features for at least 3 months who received 2 mg daily of clonazepam for 2 months (27). Subjects reported a significant relief of anxiety and HPPD symptomatology with only mild symptomatology during the clonazepam treatment, suggesting the efficacy of clonazepam in these cases (27). Espiard et al. (18) presented a case report of an HPPD patient with a previous mixed intoxication with psilocybin and cannabis. Perceptual disorders appeared after a single psilocybin consumption. The subject re-experienced the symptomatology the following day during another cannabis snort. Moreover, symptomatology was recurrent daily with an attenuation after discontinuation until 6 months after he had stopped cannabis. Initially, he received amisulpride (100 mg/daily) treatment, subsequently stopped due to sedative effects. Then he started with olanzapine (5 mg/daily) which caused an exacerbation of symptomatology. Finally, he was treated with risperidone (2 mg/daily). Then, it was coadministered with sertraline (150 mg/daily) for the treatment of persisting dysphoric mood and recurrent anxiety-like symptoms. After 6 months of combination risperidone and sertraline, HPPD disappeared (18). A 33-year-old female developed an HPPD following the recreational use/abuse of LSD for a year at the age of 18 (28). Approximately, 2–3 weeks after the last drug intake, the subject developed persistent visual disturbances (a sort of *attenuated flashbacks*), like those experienced during an acute LSD intoxication, which lasted for over 13 years, with a little change in intensity and frequency. Despite the patient receiving several psychopharmacological treatments (e.g., sertraline, citalopram, fluoxetine, risperidone, etc.), only a year-long trial of lamotrigine (100–200 mg/day) improved abnormal visual perceptions. In addition, no significant cognitive deficits (i.e., memory functioning, attention span, visual-construction, and frontal-executive functioning) were detected, but an underperformance in the phasic attention. A follow-up after 19 months showed a continued improvement in attention performance. Brain magnetic resonance imaging scans, electroencephalograms, median nerve somatosensory, and visual evoked potential tests were all reported normal (28). A case report described a 36-year-old HPPD subject who experienced persistent visual disturbances after recreational use of LSD, cannabis, alcohol, and cocaine (29). The patient was initially treated with a 3-month course of clonidine without any improvement; then, he was treated with lamotrigine (200 mg daily) for a 7-month period with a significant improvement of his visual disturbances and overall mental well-being (29). A case report described a woman who experienced panic attacks and persistent “re-experience phenomena” characterized with flashbacks to the first trip with LSD. She responded to 0.5 mg daily of risperidone (30). A case report described an HPPD patient with a history of bipolar disorder, a previous consumption of LSD, cannabis, cocaine, and phencyclidine (PCP) (30). The patient, even though treated with lamotrigine, citalopram, and mirtazapine, committed suicide, after the hospitalization (31).

### Studies on the Clinical Manifestation and Psychopathology in HPPD

A web-based questionnaire study investigated users’ perceptions of the benefits/harms of hallucinogens’ intake, including the occurrence and prevalence of flashback phenomena and/or HPPD (32). Only 10% of the sample recruited reported long-term perceptual changes, which they would “rather not have but could live with,” while 1% reported changes that “drove them mad” (32). Of these, 39% reported them following the use of LSD, 11% after psilocybin, 9% after MDMA and cannabis, 4% ketamine, and 1% alcohol. Despite 22% of subjects reported having experienced a “flashback”, only 3% of subjects experienced a “negative” flashback (32). A web-based questionnaire investigated abnormal visual experiences among 3139 hallucinogens’ users (33). Each participant had their drug-use history (i.e., classical serotonergic hallucinogens, including LSD, psilocybin, dimethyltryptamine, etc.; NMDA antagonists, including ketamine and dextromethorphan; MDMA, anticholinergic-containing Datura plants; cannabis; *Salvia Divinorum*, etc.); psychiatric and neurological history; and their visual experiences investigated. Several drugs with hallucinogenic properties have been statistically associated with unusual visual experiences, even though LSD seemed to be the most frequently reported among the HPPD-like cases (33). Lerner et al. (19) reported two case reports with a prior history of LSD intake, who experienced new visual disturbances, not previously appeared during the first LSD intoxication, after totally stopping substance use (34). A pilot study recruited 26 inpatients affected with schizophrenia and concomitant self-reported past LSD use who presented with HPPD (*n* = 12) or without HPPD (*n* = 14) (35). No significant differences in demographic, clinical features, adverse effects, and response to medications have been reported between the two groups. Furthermore, the authors reported that patients with schizophrenia and concomitant HPPD were able to distinguish HPPD symptoms from hallucinations related to their own psychotic state (35). A case-control study recruited a cannabis-user who developed HPPD and four healthy controls in order to evaluate the differences in their ophthalmological state (36). Ophthalmological examination did not report clinically significant abnormal values for the HPPD patient or for any healthy controls, even though the HPPD subject reported a slightly reduced fast oscillation rate, a diminished standing potential of the slow oscillations, and a light peak within normal range resulting in higher Arden ratios (36). Another study by Lev-Ran et al. (37) compared the characteristics of schizophrenic patients with a prior LSD consumption who developed HPPD (*n* = 37) with those who did not develop HPPD (*n* = 43). No significant differences in sociodemographic characteristics were reported between the two groups. Individuals with schizophrenia and HPPD showed lower scores for negative symptoms (*p* < 0.001), general psychopathology (*p* = 0.02), and total symptoms (*p* < 0.001), as measured with Positive and Negative Symptomatology Scale (PANSS), as compared with those reported in the group of schizophrenics without HPPD (37). A case report described a patient with a prior history of cannabis, alcohol, and LSD sporadic recreational consumption, who developed LSD-induced “Alice in Wonderland Syndrome” and HPPD after discontinuation of all substances (38). A questionnaire specifically identified prevalence and characteristics of self-reported altered perception experiences in hallucinogens’ users (39). The survey concluded that HPPD may be due to a subtle over-activation of predominantly neural visual pathways which in turn may worsen anxiety after ingestion of arousal-altering drugs (39). A study explored triggers associated with type-1 and type-2 HPPD following the use of LSD, by recruiting 40 outpatients affected with HPPD (40). The findings reported differences in terms of visual disturbances and triggers between the two HPPD types. The most common types of visual disturbances were slow movement of still objects (among type-1 HPPD) and trailing phenomena (among type-2 HPPD). Furthermore, type-1 HPPD subjects were more likely to experience disturbances in a dark environment (40).

### Studies on NPS and HPPD

A case series described two cases of HPPD induced by SCs (19). A recent case report described a patient with a previous history of heavy daily use of cannabis admitted to an Addiction Treatment Unit who reexperienced visual hallucinations and disturbances, like those who experienced during acute consumption of SC, in the days following SC consumption (20).

## Discussion

Several case reports of reoccurring or prolonged persistent visual perceptual disturbances (HPPD) have been described occurring within a certain time frame after cessation of some hallucinogenic drugs (2, 53, 54).

The present critical mini review presents several limitations. First, given the paucity of double-blind, placebo-controlled/case-control studies, we included case reports and case series as well, and studies with small sample size which may greatly limit the generalizability of findings reviewed here. Second, methodological strategies (sample size, study design, diagnostic criteria, etc.) may vary greatly in several studies retrieved. Most studies included here investigated HPPD cases following the intake of LSD, even though other isolated cases described incidents following the intake of other serotonergic hallucinogens, and cannabis. Furthermore, most studies here retrieved do not specifically distinguish between the two types of HPPD, as previously discussed, by limiting the complete understanding of the clinical symptomatology and manifestation.

The recent wave of novel substances available on the market, includes several novel cannabimimetics, novel hallucinogens (e.g., NBOMe compounds), and new LSD derivatives (48, 49). With regard to these substances, the prevalence of HPPD among NPS users is difficult to detect, due to the limited number of published reports, mainly due to the lack of knowledge among the clinicians and the consequent difficulty in the diagnosis (48). However, the specific pharmacological profile of some psychostimulants and hallucinogens may likely determine an increasing number of NPS-induced HPPD cases (48, 49). In fact, despite several studies reporting a higher occurrence of HPPD following the consumption of LSD, HPPD has been associated with a broader range of substances, e.g., natural and synthetic serotonergic 5-HT_2A_ receptor hallucinogens (44, 47, 55–57), including the most recent new synthetic psychostimulants and hallucinogens (19, 20,48, 49). In particular, the intake of SCs, which are CB_1_ receptor full agonists and possess indole-derived structures, which may itself facilitates the 5-HT_2A_ receptor dysfunction (48, 58, 59), has been associated with the onset of perceptual disorders, even after a total discontinuation, as it has been supposed that SCs may provoke a partial/total reminiscence of the previous perceptual experience in predisposed and susceptible NPS users (20, 60, 61).

Furthermore, the pharmacotherapy of HPPD may be different from study to study, as only few studies have been published so far and any recommendations are based almost entirely on non-controlled studies of small patient populations or single case reports (3, 18–31). Risperidone is a highly potent antagonist of both postsynaptic 5-HT_2_ and dopamine D_2_ receptors (62). As 5-HT_2_ receptor antagonists are effective in treating hallucinations among schizophrenic patients, it has been previously supposed that risperidone would be efficacious in treating HPPD (18, 30). However, most studies reviewed here reported a worsening of symptomatology, particularly visual disturbances and an abrupt onset of panic attacks after the intake of risperidone among LSD users (21, 28). The symptomatology tended to return to baseline levels when risperidone was discontinued (21, 28). A case report described an improvement in HPPD symptomatology with a combination of sertraline and risperidone (18). Some studies described a mild efficacy of clonidine (24, 25) or completely ineffective (28). Several studies suggested prescribing clonazepam as an effective treatment in reducing persistent perceptual disturbances (19, 20, 25, 27). In fact, it has been hypothesized that high-potency benzodiazepines (e.g., clonazepam), which as well as possessing serotoninergic activity, may be superior to low-potency benzodiazepines (27). Moreover, a case report suggested that reboxetine made a good improvement both in visual disturbances and depressive symptomatology (26). Reboxetine is an α-2-adrenoceptor modulating the effect on both noradrenaline and serotonin release which may affect sympathetic activity, hence facilitating the improvement of HPPD symptomatology (26). Other studies suggest lamotrigine as efficacious in ameliorating HPPD symptomatology (28, 29). Lamotrigine acts by blocking sodium and voltage-gated calcium channels and inhibiting glutamate-mediated excitatory neurotransmission, thereby suggesting its potential use in the treatment of HPPD (28).

Serotonin neurotransmission has been hypothesized to be involved in the aetiopathogenesys of both acute and persisting LSD- and SC-induced perceptual disturbances (61). The main mechanism supposed to be implicated consists of a vulnerability/predisposition of psychedelics’ consumers to continue centrally processing visual imagery after the visualization has been totally eradicated from the visual field (23). Persisting visual disorders may be explained by a reversible (or irreversible) “dysfunction” in the cortical serotonergic inhibitory inter-neurons with GABA-ergic outputs (63). The anandamidergic system has been also implicated by involving the areas of visual information processing (64, 65).

Further studies should be implemented in order to better clarify the role of the NPS, particularly the new psychedelics and psychostimulants with LSD-like properties in the pathogenesis and etiology of new HPPD cases.

## Author Contributions

LO and FS conceived the topic of the manuscript, while LO, AG and GP carried out the main analysis. JC and DB assisted in either screening of the studies or preparation of the attachments. FS served as study reviewer. FS served as senior study reviewer. All the coauthors substantially contributed to the present piece of work before approving it for final submission.

## Conflict of Interest Statement

The authors declare that the research was conducted in the absence of any commercial or financial relationships that could be construed as potential conflicts of interest.
